# An Acenocoumarol Dosing Algorithm Using Clinical and Pharmacogenetic Data in Spanish Patients with Thromboembolic Disease

**DOI:** 10.1371/journal.pone.0041360

**Published:** 2012-07-20

**Authors:** Alberto M. Borobia, Rubin Lubomirov, Elena Ramírez, Alicia Lorenzo, Armando Campos, Raul Muñoz-Romo, Carmen Fernández-Capitán, Jesús Frías, Antonio J. Carcas

**Affiliations:** 1 Clinical Pharmacology Service, La Paz University Hospital, Pharmacology Department, School of Medicine, Universidad Autónoma de Madrid, IdiPAZ, Madrid, Spain; 2 Pharmacology Department, School of Medicine, Universidad Autónoma de Madrid, Madrid, Spain; 3 Internal Medicine Department, La Paz University Hospital, Madrid, Spain; 4 Clinical Pharmacology Service, La Paz University Hospital, Pharmacology Department, School of Medicine, Universidad Autónoma de Madrid, Madrid, Spain; 5 Internal Medicine Department, La Paz University Hospital, School of Medicine, Universidad Autónoma de Madrid, Madrid, Spain; College of Pharmacy, University of Florida, United States of America

## Abstract

Appropriate dosing of coumarins is difficult to establish, due to significant inter-individual variability in the dose required to obtain stable anticoagulation. Several genetic and other clinical factors have been associated with the coumarins dose, and some pharmacogenetic-guided dosing algorithms for warfarin and acenocoumarol have been developed for mixed populations. We recruited 147 patients with thromboembolic disease who were on stable doses and with an international normalized ratio (INR) between 2 and 3. We ascertained the influence of clinical and genetic variables on the stable acenocoumarol dose by multiple linear regression analysis in a derivation cohort (DC; n = 117) and developed an algorithm for dosing that included clinical factors (age, body mass index and concomitant drugs) and genetic variations of *VKORC1*, *CYP2C9*, *CYP4F2* and *APOE*. For purposes of comparison, a model including only clinical data was created. The clinical factors explained 22% of the dose variability, which increased to 60.6% when pharmacogenetic information was included (p<0.001); *CYP4F2* and *APOE* variants explained 4.9% of this variability. The mean absolute error of the predicted acenocoumarol dose (mg/week) obtained with the pharmacogenetic algorithm was 3.63 vs. 5.08 mg/week with the clinical algorithm (95% CI: 0.88 to 2.04). In the testing cohort (n = 30), clinical factors explained a mere 7% of the dose variability, compared to 39% explained by the pharmacogenetic algorithm. Considering a more clinically relevant parameter, the pharmacogenetic algorithm correctly predicted the real stable dose in 59.8% of the cases (DC) vs. only 37.6% predicted by the clinical algorithm (95% CI: 10 to 35). Therefore the number of patients needed to genotype to avoid one over- or under-dosing was estimated to be 5.

## Introduction

Coumarin anticoagulants, including warfarin, acenocoumarol and phenprocoumon, are highly effective antithrombotic drugs for the treatment of thromboembolic diseases (such as deep venous thrombosis and pulmonary embolism), atrial fibrillation and artificial heart valves [1]. Appropriate dosing of coumarins is difficult to establish, due to widespread inter-individual variability in its pharmacokinetics and pharmacodynamic responses and its narrow therapeutic window. Numerous genetic and non-genetic factors have been associated with the inter-individual variability in warfarin and acenocoumarol dosing requirements. Although warfarin and acenocoumarol are very similar, the recommended doses are different and they have differences in their pharmacokinetics and pharmacodynamics, as well as in the influence of genetics and other factors [2]. Thus, the effect of CYP2C9 defective variants is more pronounced for warfarin than for acenocoumarol and as a consequence the explained variability in dosing is 10–15% in the case of warfarin and about 5% in the case of acenocoumarol [3]. Therefore, data on the influence of pharmacogenetics and other factors on warfarin dose (the most well-studied to date) to obtain stable anticoagulation are not applicable to acenocoumarol or phencroumon.

Acenocoumarol, a derivative of coumarin, is commonly used in Spain and other European countries (France, Ireland, UK, Netherlands, Austria, Belgium, France, Italy, Portugal, Poland, Switzerland and Hungary) and numerous countries around the world (Argentina, Chile, India, Ukraine, Israel, Mexico and Canada) [4]. Presently, the initial dosing of acenocoumarol is based mainly on demographic and clinical characteristics, and later dosing is based on INR results obtained during subsequent days and weeks. However, there is substantial variability in this approach, based on the experience and background of the attending physician. In our Autonomous Community (Madrid) there are consensus guidelines for oral anticoagulation applicable to the Regional Health System. Typically, patients start with daily doses of about 2 mg (1 mg in elderly patients), and thereafter the dose is adjusted in accordance with INR test results. Numerous factors have been associated with the acenocoumarol dose required to obtain stable anticoagulation, including age, gender, weight, height, drug interactions and variations in the *VKORC1* and *CYP2C9* genes [5]–[6]. Other influential genes, such as *CYP4F2* [7]–[10] and *APOE* [11]–[12], have been identified, but their roles are controversial. Teicher et al. [9] described the minor influence of *CYP4F2* in a genome-wide association study of a prospective cohort designed to study neurological, cardiovascular, locomotor and ophthalmological diseases in a population aged 55 years or older [13]. However, Perez-Andreu et al. [10] have suggested that *CYP4F2 V433M* may play an important role in patients with high dosing requirements. In the case of *APOE*, only Visser et al. [11] observed an association between the ε4 allele and the required doses of acenocoumarol.

Given the high rate of adverse effects (including fatal hemorrhagic events [14]–[15]) due to incorrectly calculated doses, it is necessary to develop new strategies for determining the appropriate weekly dose of acenocoumarol. Several pharmacogenetic algorithms for predicting an appropriate warfarin dose have been proposed [16]–[25]. Two dosing algorithms for acenocoumarol have been published. The first [26] was based on 193 outpatients on stable anticoagulation. The authors constructed an “acenocoumarol-dose genotype score” based on the number of alleles associated with higher acenocoumarol dosage carried by each subject for each polymorphism, in order to predict those patients who would require high acenocoumarol doses to achieve stable anticoagulation. The second was published recently [27], and included patients on phenprocoumon (n = 229) and acenocoumarol (n = 168) treatment. The pharmacogenetic information included VKORC1 and CYP2C9 genotypes, in addition to clinical information (weight, height, sex, age and amiodarone). Their algorithm explained 52.6% of the variance in the acenocoumarol maintenance dose in the derivation cohort and 49% in the validation cohort. In both cases the population included patients with a wide range of indications: atrial fibrillation, cardiac valve replacement, thromboembolic disease, and other conditions.

After the development of a pharmacogenetic algorithm, the natural next step is to demonstrate its effectiveness and utility by mean of a controlled randomized trial. One such trial is being carried out with warfarin in the USA: NCT01178034 (ClinicalTrial.gov) including patients with atrial fibrillation. Another trial has been completed: NCT00511173, including patients with atrial fibrillation, pulmonary embolism and deep vein thrombosis. In Europe, a clinical trial is ongoing to test whether the dosing algorithms for coumarin anticoagulants (including acenocoumarol) improve the clinical outcomes of patients [28].

The aim of this study was to develop and validate a pharmacogenetic dosing algorithm for acenocoumarol in a well-defined cohort of patients with thromboembolic disease. We also assessed the algorithm's performance when compared to only demographic and clinical factors, to evaluate its potential clinical relevance. This dosing algorithm is currently being tested in a randomized trial (Eudra CT: 2009-016643-18).

## Materials and Methods

### Design and setting

This was an observational, prospective, transversal study. Patients attending the Thromboembolic Disease Unit of the Internal Medicine Service of La Paz University Hospital (Madrid, Spain) between 04/2008 and 06/2009 and meeting the selection criteria were proposed to participate in the study. Ethical permission for this study was obtained from the Clinical Research Ethics Committee of La Paz University Hospital of Madrid, Spain

### Patients and data collection

The main inclusion criteria were as follows: patients with a diagnosis of pulmonary embolism and/or deep venous thrombosis receiving a stable dose of acenocoumarol (weekly dose variation <20% in the last three months) and an INR within the range of 2 to 3 for at least the three previous months. In order to include patients with occasional INR out of this therapeutic range of 2–3, an occasional INR in the range of 1.8 to 3.5 was allowed. These limits were chosen considering that typically, an acenocoumarol dose variation below 10% is recommended if INR values are in this wider range. Exclusion criteria included renal (calculated creatinine clearance <30 ml/min), hepatic (Child-Pugh stage C) or thyroid dysfunction and cancer.

All patient data and blood samples for *CYP2C9*, *VKORC1*, *CYP4F2* and *APOE* genotyping were collected during a visit to the clinic after written informed consent was obtained. Patient data collected included: age, gender, race, body weight and height (and calculated BMI), smoking status, INR results and acenocoumarol dose administered in the last 3 months, patient's education level and concomitant medications. A mini-mental test was also performed and recorded.

### Genotyping

Blood samples were collected in tubes containing EDTA (ethylenediaminetetraacetic acid) and stored at −20°C before extraction. DNA was isolated using the QuickGene DNA blood kit S (Fujifilm®, Düsseldorf, Germany). KASPar® technology (KBiosciences©, Hoddesdon, UK) was used to detect the CYP2C9*3 (rs1057910), *CYP4F2* (rs2108622), *VKORC1* (−1639 G→A = rs9923231) and *APOE* (8016 C→T = rs7412 and 7878 T→C = rs429358) variant alleles. Taqman® technology (Applied Biosystems©, Foster City, USA) was used to detect CYP2C9*2 (rs1799853). The Hardy-Weinberg equilibrium was calculated with STP-Analyzer 1.2A (Istech, INC®).

### Algorithm generation and evaluation of bias and precision

We randomly chose 80% of the included patients (stratified according to *CYP2C9* genotype nested to *VKORC1 A/A* vs. *VKORC1 A/G* and *GG*) as the “derivation cohort” (DC) for developing the dose-prediction model. The remaining 20% of the patients constituted the “test cohort” (TC), which was used to test the final model selected.

After preliminary analysis using univariate and various multivariate methods and a review of methods used in the literature, multiple linear regression (MLR) was used to derive the predictive model. The dependent variable used was the dose needed to obtain a stable INR (ln-transformed), and as independent variables we included all the demographic, clinical and genotypic factors collected (see Table S1).

We performed preliminary MLR using the entire and the derivation cohorts; the analysis included the “introducing” method, the “backward” step method and the “forward” step methods available in SPSS. The results are summarized in Table S2. After this process we choose the variables to be included in the model, selecting those variables found with p-values consistently below 0.1 as shown in Table S2. After running the MLR with these variables, the final model (pharmacogenetic algorithm) was determined. For comparison, a clinical algorithm was also built, using only the clinical independent variables used in the previous model. The p- and β values are provided, reflecting the significance and the adjusted relative weight, respectively, of each variable included in the model.

The performance of the pharmacogenetic and clinical models was evaluated initially in three cohorts: entire, derivation and test. The calculated uncorrected coefficient of determination (*R^2^*) of each model shows the total variability explained by the model. To ascertain the contribution of each group of variables to the final model, we calculated the unadjusted R^2^, including only the clinical variables, and consecutively the rest of the variables in the model.

After back transformation of the dose predicted by the models, we calculated the mean error (ME; mean of the differences between predicted and observed acenocoumarol doses), mean absolute error (MAE; absolute difference between predicted and observed acenocoumarol doses) and the ME and MAE expressed as a percentage of the observed acenocoumarol dose (%ME and %MAE). For these parameters, standard deviation and 95% confidence intervals were also calculated. The ME reflects the bias in the prediction, and MAE is an estimation of the precision of the model [29].

All analyses were performed using SPSS 16.0 (Inc., IL, USA).

### Clinical relevance

To evaluate the clinical relevance of the models built, we classified the patients into three dose groups: patients requiring a low dose (<25^th^ percentile; ≤11 mg/week), those requiring a high dose (>75^th^ percentile; ≥21 mg/week) and those requiring intermediate doses (25^th^ to 75^th^ percentile). Next, we calculated the percentage of patients for whom the predicted dose was within ±20% of the real stable dose of acenocoumarol.

To evaluate the potential benefit of using the pharmacogenetic algorithm, we calculated the number needed to genotype (NNG) as used by the International Warfarin Pharmacogenetics Consortium and defined as the number needed to genotype to avoid misclassifying one patient (into one of the predefined dose groups) by the pharmacogenetic model in comparison with the clinical model [21]. The NNG was computed using the standard “number needed to treat” method [30]. The NNG is the inverse of the absolute risk reduction (ARR), calculated as the absolute difference between the event rates for the pharmacogenetic and clinical algorithms.

## Results

### Patient characteristics

A total of 147 Caucasian patients participated in this study (entire cohort -EC-), 117 in the “derivation cohort” (DC) and 30 in the “test cohort” (TC). The patients' demographics, genotypes and concurrent medications are shown in Table 1. No statistical differences were observed between the derivation and test cohorts. No patients on amiodarone or enzyme inducers, or carrying *APOE* rs7412 fell in the test cohort. Each SNP was in Hardy-Weinberg equilibrium.

**Table 1:** Characteristics of study cohorts.

Variable	Derivation cohort	Testing cohort	P value
	(N = 117)	(N = 30)	
Gender [n (%)]			0.29
Male	61 (52.1)	14 (46.7)	
Female	56 (47.9)	16 (53.3)	
Age, in years [mean (SD)]	67.6 (17)	67.5 (17.7)	0.73
Weight, in kilograms [mean (SD)]	74.3 (15.4)	75.5 (13.5)	0.59
Height, in meters [mean (SD)]	1.63 (0.1)	1.62 (0.1)	0.9
Body mass index (BMI), in kg/m2 [mean (SD)]	27.8 (4.7)	28.6 (4.3)	0.66
Current smoker [n (%)]	12 (10.3)	5 (16.7)	0.96
Mini-mental test [mean (SD)]	27.0 (4.0)	27.0 (3.6)	0.56
Acenocoumarol weekly dose [mean (SD)]	16.7 (7.4)	15.7 (6.0)	0.46
Patients' education			0.83
No education	14 (12.2)	6 (20.0)	
Primary school	55 (47.8)	14 (46.6)	
Secondary school	29 (25.2)	6 (20.0)	
University degree	17 (14.8)	4 (13.4)	
Concurrent medications [n (%)]			
Enzyme inducers(1)	5 (4.3)	0	0.25
Enzyme inhibitors(2)	63 (54.7)	16 (53.3)	1.00
Amiodarone	2 (1.7)	0	0.47
Non-steroidal anti-inflammatory drug	17 (14.8)	1 (3.3)	0.12
*CYP2C9* genotype [n (%)] (3)			0.62
*1/*1	60 (51.7)	19 (63.3)	
*1/*2	37 (31.9)	6 (20)	
*1/*3	16 (13.8)	4 (13.3)	
*2/*2 or *2/*3 or *3/*3	3 (2.6)	1 (3.3)	
*VKORC1* genotype [n (%)]			0.34
G/G	49 (41.9)	9 (30)	
A/G	49 (41.9)	17 (56.7)	
A/A	19 (16.2)	4 (13.3)	
*CYP4F2* genotype [n (%)](4)			0.24
VV	46 (40.4)	15 (55.6)	
VM	54 (47.4)	8 (29.6)	
MM	14 (12.3)	4 (14.8)	
*APOE* rs7412 genotype [n (%)]			0.133
C/C	103 (88.0)	23 (76.7)	
C/T	12 (10.3)	7 (23.3)	
T/T	2 (1.7)	0	
*APOE* rs429358 genotype [n (%)]			0.551
T/T	94 (82.5)	20 (76.9)	
T/C	18(15.8)	6 (23.1)	
C/C	2 (1.7)	0	

(1) *CYP* inducers that were considered in this analysis included phenytoin, carbamazepine and rifampin.

(2) *CYP* inhibitors that were considered in this analysis included azoles, proton pump inhibitors and statins.

(3) For *CYP2C9* genotype, the usual * designation is used (*2 = rs1799853 and *3 = rs1057910).

(4) VV indicates homozygous V433 carriers; VM, heterozygous V433M carriers; MM, homozygous M433 carriers.

### Clinical and pharmacogenetic acenocoumarol dose algorithms

For both the pharmacogenetic and clinical algorithms, Table 2 shows the clinical, demographic and genotypic independent variables that were ultimately included in the multiple linear regression analysis, as well as those variables that were considered the best at predicting the weekly doses of acenocoumarol needed to obtain a stable INR. β values and the significance level of each variable included in the model are also provided in this table. Table 3 shows the variability explained by clinical factors, *CYP2C9*, *VKORC1*, *CYP4F2* and *APOE* in the pharmacogenetic algorithm. *CYP4F2* and *APOE* together explain 4.9% of the variability. The variability explained by the models (R^2^) was 60.6% for the pharmacogenetic algorithm and 22.0% for the clinical algorithm (Table 4). This difference is statistically significant (p<0.001; McNemar's test of paired proportions).

**Table 2:** Variables ultimately included in the acenocoumarol pharmacogenetic and clinical dosing algorithms, and values of Beta reflecting their relative weight in the final model.

Pharmacogenetic algorithm			Clinical algorithm		
Beta	Variable	P value	Beta	Variable	P value
**Clinical variables**					
−0.294	Age	<0.0001	−0.01	Age	0.001
0.240	BMI	<0.0001	0.016	BMI	0.053
0.119	Enzyme inducer status	0.062	0.41	Enzyme inducer status	0.022
−0.142	Amiodarone status	0.026	−0.45	Amiodarone status	0.13
**CYP2C9**					
−0.257	CYP2C9 *1/*3	<0.0001			
−0.253	CYP2C9 *2/*2 or *2/*3 or *3/*3	<0.0001			
**VKORC1**					
−0.150	VKORC1 A/G	0.039			
−0.533	VKORC1 A/A	<0.0001			
**CYP4F2**					
0.199	CYP4F2 MM	0.002			
**APOE**					
0.123	APOE (rs7412)T/T	0.067			

Beta: standardized regression coefficient, which reflects the relative weight of each variable included in the model.

**Table 3:** Unadjusted R^2^ for each group of variables and resultant cumulative R^2^ of the final model.

Pharmacogenetic algorithm	
	Variable	R^2^(%)	Cumulative R^2^(%)
**Clinical variables**		22.0%	22.0%
	Age		
	BMI		
	Enzyme inducers status		
	Amiodarone status		
**CYP2C9**		11.7%	33.7%
	CYP2C9 *1/*3		
	CYP2C9 *2/*2 or *2/*3 or *3/*3		
**VKORC1**		22.0%	55.7%
	VKORC1 A/G		
	VKORC1 A/A		
**CYP4F2**		3.6%	59.3%
	CYP4F2 MM		
**APOE**		1.3%	60.6%
	APOE (rs7412)T/T		

**Table 4:** Predictive performance of pharmacogenetic and clinical algorithms.

	Pharmacogenetic algorithm	Clinical algorithm	Difference (95% CI)	P value
**Derivation cohort (n = 117)**				
R2‡	60.6%‡	22.0%		<0.001
ME	−0.66 (5.01)	−1.22 (6.68)	**−0.55**	0.142
			(−1.29 to 0.18)	
MAE	3.63 (3.50)	5.08 (4.48)	**1.46**	<0.001
			(0.88 to 2.04)	
%ME	4.43 (33.59)	8.92 (50.20)	**4.49**	0.212
			(−2.60 to 11.58)	
%MAE	23.43 (24.38)	34.53 (37.38)	**11.09**	<0.001
			(5.04 to 17.16)	
**Testing cohort (n = 30)**				
R2‡	38.8%‡	7.0%		<0.001
ME	0.31 (4.99)	−0.13 (5.87)	**0.43**	0.554
			(−1.91 to 1.05)	
MAE	3.75 (3.24)	4.86 (3.18)	**1.11**	0.083
			(−0.16 to 2.37)	
%ME	9.96 (34.63)	12.08 (45.76)	**2.11**	0.761
			(−11.97 to 16.20)	
%MAE	25.76 (24.81)	35.05 (31.21)	**9.29**	0.138
			(−3.16 to 21.74)	
**Entire cohort (n = 147)**				
R2‡	56.8%‡	19.0%		<0.001
ME	−0.46 (5.00)	−0.99 (6.52)	**−0.53**	0.113
			(−1.18 to 0.13)	
MAE	3.65 (3.44)	5.03 (4.23)	**1.38**	<0.001
			(0.86 to 1.91)	
%ME	5.57 (33.76)	9.57 (49.18)	**4.00**	0.208
			(−2.26 to 10.26)	
%MAE	23.9 (24.40)	34.64 (36.09)	**10.72**	<0.001
			(5.34 to 16.11)	

ME: mean error (predicted – observed); %ME: mean error expressed as a percentage (%ME = ME/Observed*100); MAE: mean absolute error ( = SQR[(Pred-Obs)^2^]); %MAE: mean absolute error expressed as a percentage (%MAE = MAE/Obs*100).

‡ McNemar's test of paired proportions.

### Bias and precision of pharmacogenetic and clinical algorithms in the cohorts

Bias (ME and %ME) and Precision (MAE and %MAE) of the pharmacogenetics model was very similar in the derivation and test cohorts and therefore in the entire cohort (Table 4). Bias was very low: −0.66 (SD 5.01), 0.31 (SD 4.99) and −0.46 (SD 5.00) in the DC, TC and EC respectively. Precision is a more clinically relevant parameter that was also quite low and very similar between cohorts; thus the weekly predicted dose deviates from the actual dose by 3.63 mg (±3.50) in the DC, by 3.75 mg (±3.24) in the TC and by 3.65 mg (±3.44) in the EC. These differences were not statistically significant and therefore we can conclude that the model behaves similarly in the three cohorts.

On the other hand, the performance of the clinical algorithm is clearly poorer. First, as previously stated, the variability explained by the clinical variables alone is lower than that obtained by the pharmacogenetic algorithm: 22.0% versus 60.6% in the DC, 7% vs. 38.8% in the TC, and 19.0% vs. 56.8% in the EC, with all differences being statistically significant (see Table 4). Also MAE (and %MAE) is significantly higher in the clinical algorithm when compared to the pharmacogenetic algorithm in both derivation and entire cohorts, although the difference only approaches statistical significance in the test cohort (p = 0.083 for MAE) due to its lower size.

From a clinical point of view, a calculation of the percentage of patients correctly classified within the ±20% of the real dose obtaining a stable INR would be more relevant. The pharmacogenetic algorithm correctly predicts the weekly acenocumarol dose in 57.1% of the EC patients, 59.8% of DC cases, and 46.7% in the TC cohort. The figures for the clinical algorithm are lower: 34.7%, 37.6% and 23.3%, respectively (see Table 5).

**Table 5:** Percentage of global correct classification (Predicted Dose within ±20% of Real Dose) by genetic and clinical algorithms in the derivation, test and entire cohorts.

	% correctly classified		ARR (95% CI)	NNG (95% CI)
	Pharmacogenetic	Clinical		
Derivation cohort (n = 117)	70/117	44/117	22.0%	4.5
	59.8%	37.6%	(10 to 35)*	(2.88 to 10.27)
Testing cohort (n = 30)	14/30	7/30	23.3%	4.3
	46,7%	23,3%	(0.0 to 47)	(−2.14 to 1359)
Entire cohort (n = 147)	84/147	51/147	22.0%	4.5
	57.1%	34.7%	(11 to 34)*	(2.98 to 8.81)

* p<0.001

### Performance of pharmacogenetic and clinical algorithms by dose group

As has been shown by other authors [5], [6], [10], [21], most of the incorrectly dosed patients when using standard initiation doses are those needing low or high Coumadin doses, and therefore it is important to analyze the performance of the algorithm in different dose subgroups. Table 6 shows the differences in precision (in absolute terms) between the pharmacogenetic and clinical algorithms in the preformed dose groups. Complete data including bias and precision in the three cohorts are shown in Table S3. In the EC, differences in bias and precision parameters between pharmacogenetic and clinical models are statistically significant in the low and high dose groups, but do not consistently reach significance in the intermediate group. In the low dose group, the dose predicted by the clinical algorithm overestimates the actual dose by 60.60%; however the dose overestimation of the pharmacogenetic algorithm is limited to 37.38% (p = 0.005). In the high dose group, both algorithms underestimate the dose but to a lesser degree in the pharmacogenetic algorithm: 22.45%, versus 31.60% in the clinical algorithm (p<0.001). Bias and precision in the DC is quite similar to that observed in the EC. In the TC, differences only approach statistical significance in the high dose group (see Table 6 and Table S3).

**Table 6:** Precision expressed as MAE (SD) of pharmacogenetic and clinical algorithms by dose group in the entire cohort.

Dose Group	PhGx algorithm	Clinical Algorithm	Difference	P value*
**Low (n = 46)**				
MAE	3.36 (3.13)	4.95 (3.30)	1.59 (3.84)	0.008
			0.44 to 2.75	
% correctly classified	41%	13%	29 (11 to 46)	0.0049
**Median (n = 62)**				
MAE	2.28 (2.04)	2.81 (2.49)	0.52 (2.43)	0.099
			−0.10 to 1.14	
% correctly classified	77%	61%	16 (0 to 32)	0.051
**High (n = 39)**				
MAE	6.13 (4.18)	8.62 (4.93)	2.49 (3.08)	<0.001
			1.50 to 3.49	
% correctly classified	44%	18%	26 (6 to 45)	0.0272

* Between-group comparisons calculated by paired “t” test.

Table 6 also shows a comparison of the percentage of well-classified patients in the three pre-established dose groups, as determined by both the pharmacogenetic and clinical models. The pharmacogenetic algorithm correctly predicts the actual dose for a higher percentage of patients than the clinical algorithm. For those patients requiring a low dose, the pharmacogenetic algorithm provides significantly better prediction of the dose than the clinical algorithm: 41% vs. 13%, (absolute difference: 29%; 95% CI: 11–46). Similarly, for patients requiring a high dose, the pharmacogenetic algorithm performs significantly better than the clinical algorithm: 44% vs. 18% (absolute difference: 26%; 95% CI: 6–45). For patients requiring an intermediate dose, the differences are less and statistical significance is marginal: 77% correct prediction by the pharmacogenetic algorithm vs. 61% by the clinical algorithm (absolute difference: 16%; 95% CI: 0–32).

To globally estimate the potential clinical relevance of the pharmacogenetic algorithm, we calculated the number of patients needed to genotype (NNG) to avoid over- or under-dosing (i.e., misclassifying patients). The global percentage of correctly classified doses by the pharmacogenetic algorithm in the entire cohort was 57.1% compared with 36.7% when using only clinical variables. Therefore the absolute difference is 22% and the NNG is 4.5 (95% CI: 3–8.8). The figures are similar for the derivation and test cohort. Table 5 depicts the percentage of patients misclassified by the pharmacogenetic and clinical algorithms, the absolute risk reduction (ARR) between both algorithms and the calculated NNG with their corresponding confidence intervals. In all cases, the ARR is about 20% and therefore the NNG about 5, although in the test cohort neither reached statistical significance.

## Discussion

Oral anticoagulation with coumarin derivatives is associated with a high incidence of bleeding complications as well as therapeutic failures. Every year, 2–5% of patients on anticoagulant therapy experience serious bleeding, and 0.5–1% of patients have a fatal bleeding episode [1]. These complications are due to the narrow therapeutic range of the INR and to the high dose variability needed to obtain stable anticoagulation, which is reached after many INR checks and changes to the dose based on trial and error. Thus, according to Caraco et al., the time needed to obtain a pharmacodynamic steady state in warfarin-treated patients is 40.27 days (95% CI, 35.9–44.6 days) [31]. For acenocoumarol, Gadisseur et al. [32] report that patients are maintained in the therapeutic range only 30% of the time during the first 6 weeks of treatment. This lengthy method to obtain an efficacious and stable INR carries the risk of inefficacy and increases bleeding episodes in the first month of anticoagulation, as described by Landefeld et al. [33]. Also, the time within the therapeutic range strongly correlates with bleeding and the rates of thromboembolism [34]–[35].

One potential approach to improving anticoagulant treatment would be to use a dosing algorithm including demographic and clinical variables and genetic testing. Some algorithms have been published for warfarin [16]–[18], [23]–[25] but only a few for acenocoumarol [26], [27]. Some of these algorithms are in the process of validation through clinical trials, as previously mentioned. The population included in this type of study is usually a broad-spectrum population that mainly includes patients with atrial fibrillation, prosthetic valves and thromboembolic disease. On the other hand, the sources of the patients are registries from hematology and anticoagulation clinics and therefore are based on retrospective data.

In this article, we describe the development and performance of a pharmacogenetic algorithm for acenocoumarol dosing in a prospective cohort of patients with thromboembolic disease. These patients are typically younger, have fewer concurrent conditions [36] and have a lower target INR (2 to 3). Clearly, this implies the theoretical advantage of less variability in the population characteristics and target INR. A drawback is that application of our algorithm may be limited mainly to patients with DVT and PE, and its use in other patients will require prior validation. However, it must be stressed that in thromboembolic disease it is crucial to obtain the target INR as soon as possible to avoid a lack of efficacy as well as excessive coagulation.

Another characteristic of this study is that it is the first acenocoumarol algorithm including *CYP4F2* and *APOE* in addition to the well-known *VKORC1* and *CYP2C9* gene variants, as well as clinical factors such as age, BMI and interacting drugs. All together these factors in our model explain 60.6% of the total variability in the acenocoumarol dose needed to obtain a stable INR. This percentage is significantly higher compared to the variability explained when only non-genetic factors are considered. This explained variability is similar or better than that obtained in other studies with warfarin [21], [23]–[25] and acenocoumarol [26], [27]. The contribution of *CYP4F2* and *APOE* to the explained variability in the acenocoumarol dose by our algorithm is 3.6% and 1.3%, respectively.

The influence of *CYP4F2* has been described in several studies with warfarin and acenocoumarol and has only been included in a published algorithm for warfarin [23] and in the algorithm available at WarfarinDosing.org. The contribution of *CYP4F2* in our study is similar to the 4% described by Sagrieya et al. for warfarin [37] and can be considered relevant.

Apolipoprotein E (*APOE*) mediates the uptake of vitamin K-rich lipoproteins in the liver and other tissues. It is a polymorphic protein defined by three alleles, ε2, ε3 and ε4 (defined by two SNPs, rs429358 and rs7412), at a single gene locus on chromosome 19. Visser et al. [11] observed that individuals who are homozygous or heterozygous for the ε4 allele (rs429358) require significantly lower doses of acenocoumarol to reach the same level of anticoagulation as patients with the ε3/ε3 genotype, and individuals with the ε2/ε2 or ε2/ε3 (homozygous or heterozygous for rs7412, respectively) genotypes require higher doses of acenocoumarol (however, not a statistically significant difference). Also Cavallri et al. showed that *APOE* is associated with the time to achieve a stable warfarin dose in African-American patients [38]. To date, *APOE* has not been considered in any of the algorithms available. The inclusion of *APOE* in our algorithm needs discussion, as it is based on only two patients each and the statistical significance in the model is slightly above 0.05 (p = 0.067). The inclusion of this variable was based on three facts: a) all or most of the preliminary regression analyses we performed included it; b) the weight of the factor in the final model as reflected by its β value in the model was considered quite high (in the range of that observed for *VKORC1 A/G*) and c) it is associated with the need for higher doses, for which other algorithms perform more poorly. For example, the IWPG algorithm [21] only classified 24.8% of the patients needing higher doses of warfarin correctly. On the other hand, the contribution of *APOE* (1.3%) to the total variability explained by our model is low but is still considered valuable. Several algorithms have included variables contributing less to the variability and some publications have considered contributions of this magnitude to be adequate (e.g., gender and amiodarone use in the EU-PACT algorithm).

On the other hand, the inclusion of amiodarone in the model is clearly justified as its influence in the coumarins dose is clearly established in the literature.

We generated the algorithm using linear regression, which has the advantages of simplicity and ease of use. This approach has been previously used by the International Warfarin Pharmacogenetics Consortium (IWPC) [21] and by the EU-PACT Study Group [26]. Also we tested our algorithm in a different cohort that was randomly obtained from the entire cohort. Obviously, given that the test cohort is small, its R^2^ is lower than that of the derivation cohort (38.8% vs. 60.6%), but bias and precision are very similar in the test cohort and in the derivation and entire cohorts (see Table 3). By comparison, using their algorithm, the IWPC obtained a lower R^2^ for warfarin in their derivation cohort than that obtained with our algorithm (47% vs. 60.6%, respectively) and a higher R^2^ in their test cohort (43% vs. 38.8%, respectively), as their test cohort was much larger. The algorithm developed by the EU-PACT Study Group for acenocoumarol shows a slightly lower R^2^ value in the derivation cohort (n = 471): 52.6% for the genotype-guided dosing; in the validation cohort their figure was 47.3%. As shown in Table 7, we obtained similar or even better MAEs and percentages of correctly classified patients according to the actual dose than in the IWPC and EU-PACT studies. It is worth mentioning that despite the differences in the type of patients included, the sources of data and the number of patients included (in addition to the drug itself), the performance of the genetic algorithms was similar in the three studies.

**Table 7:** Comparison of R^2^ and MAE in our study and two other studies (IWPC and EU-PACT).

	IWPC (20)		EU-PACT (26)		This Study	
	EC	VC	DC	VC	DC/EC	VC
	(n = 4043)	(n = 1009)	(n = 375)	(n = 168)	(n = 117)	(n = 30)
R^2^	47%	43%	52.6%	49.0%	60.6%/56.8%	38.8%
MAE	≈4.7	≈4.7	3.64	3.99	3.63/3.65	3.75
% correctly classified	≈46%	≈45,5%	NA	NA	59.8/57.1%	46.7

DC: Derivation cohort; EC: entire cohort; VC: Validation/Testing cohort; MAE: mean absolute error (mg/week). MAE for warfarin dose has been corrected considering a dose equivalence ratio of 0.57 between both drugs.

When the actual required dose was classified into three groups, the potential of our algorithm to assign patients into the correct dose group was good. In those patients needing a standard dose (11–21 mg/week), the performance of the pharmacogenetic algorithm was slightly better than the clinical algorithm in the entire and derivation cohorts (77.0% vs. 61.0% in the EC and 78.9% vs. 61.5% in the DC) but did not reach statistical significance in the TC cohort due to the smaller size (70% vs. 60%). However, in the group requiring lower weekly doses (<11 mg/week, representing 31.3% of the cohort), the differences in the percentage of correctly classified patients were clinically relevant and significantly higher using the pharmacogenetic algorithm (41.0% vs. 13.0% in the EC, 44.1% vs. 14.7% in the DC and 33.3% vs. 8.3% in the TC). A similar result was found in the high dose group, where the percentage of correctly classified patients by the pharmacogenetic algorithm was significantly higher (44.0% vs. 18.0% in the EC, 45.2% vs. 22.6% in the DC and 37.5% vs. 0.0% in the TC). Complete data are detailed in Table S4.

Another important aspect to consider is the over- and underestimation of the predicted doses in the low and high-dose groups. In patients needing low doses, both the clinical and pharmacogenetic algorithms overestimated the dose, but this overestimation was lower with the pharmacogenetic algorithm (in the EC it was 3.36 mg/week vs. 4.95 mg/week in the clinical algorithm). The opposite phenomenon occurred in the patients needing the highest dose, for whom the better performance of the pharmacogenetic algorithm over the clinical algorithm was maintained (6.13 vs. 8.62 mg/week, as in the EC). See the supplementary material for complete data. This phenomenon was also observed in the IWPG study [21] but was not evaluated in the EU-PACT study [26]. In this aspect our algorithm seems to perform better than the IWPG algorithm when predicting doses for patients who need higher doses. Across the entire cohort, our algorithm was able to correctly classify 44% of patients in need of higher doses, compared to 26.4% in the case of the IWPG algorithm. These figures were 37.5% and 24.8%, respectively, in the validation/test cohort. This data suggest that the inclusion of the *CYP4F2* and *APOE* genotypes, may improve prediction in patients needing high doses, because these genotypes are associated with a higher dose of acenocoumarol (and probably warfarin) to obtain the target INR.

Considering the global benefits of using our acenocoumarol pharmacogenetic algorithm, the number needed to genotype to avoid a misclassified patient (NNG) can be calculated to be 5 in any cohort. This result is at least as clinically relevant as those obtained by similar studies with warfarin [21]. Therefore if dose adjustment in clinical practice behaves similar to the clinical algorithm, this pharmacogenetic algorithm could better predict the needed acenocoumarol dose and prevent patients from being over- or undercoagulated.

The main limitation of our study was the limited sample size. First, we did not have enough patients in the derivation cohort to include potentially important factors that could influence the stable dose of acenocoumarol, such as smoking status or other concurrent medications. Nevertheless, the R^2^ (60.6%) of our algorithm (the percentage of dose variability explained by the model) was higher than that of other acenocoumarol (and warfarin) dosing algorithms, indicating good performance when predicting actual doses and indicating more accurate dose predictions compared to the use of only clinical variables. This improved prediction was especially true for patients who need low or high doses and, therefore, have a higher risk of bleeding or re-thrombosis. Another limitation is the size of the testing cohort, which was too small to include every type of patient included in the derivation cohort and to give it enough power for most of the comparisons. Also this small size would explain the low R^2^ of the clinical algorithm. However, the behavior of the pharmacogenetic algorithm in this cohort is very similar to the performance in the test or entire cohort in terms of bias and precision measures and in the percentage of correctly classified patients. The limitation due to the restricted population (and target INR) has already been discussed; however, this population restriction also has the potential advantage of higher specificity, and we can hypothesize that this could be a reason for the robust performance of our model despite the low numbers.

We have not included other genes that have been described to influence coumarins dosing. Coumarins sensitivity has been also associated to polymorfirms of γ-glutamyl carboxylase (GGCX) that activates several clotting factors, and to chaperon calumenin (CALU) which inhibits GGCX, but this later polimorfirms is very rare in Caucasian and its influence in acenocoumarol dosing seems to be very low [39].

In conclusion, we have developed the first pharmacogenetic dosing algorithm for acenocoumarol that includes clinical variables and information about four genes (*VKORC1, CYP2C9, CYP4F2* and *APOE*) and that is able to reasonably predict stable therapeutic doses of acenocoumarol for patients with thromboembolic disease. It would be especially useful in clinical practice for patients requiring low (<11 mg/week) or high (>21 mg/week) therapeutic acenocoumarol doses, which represent nearly 60% of the entire cohort. To test the efficiency and effectiveness of this pharmacogenetic dosing algorithm for acenocoumarol vs. usual care, a controlled, randomized, single-blind, multi-center clinical trial has been designed and is being carried out (Eudra CT:2009-016643-18). The trial is scheduled to be completed by mid-2012.

## Supporting Information

Table S1 **Demographic, clinical and genotype data in the model.**
(DOCX)

Table S2 **Results of Multiple Linear Regresion (LR) in the Entire Cohort (EC) and Derivation Cohort (DC) following different methods for variable evaluation (Introduce, by forward steps and backward steps).** After these analyses the variables to be included in the final model were selected as depicted in the last column.
(DOCX)

Table S3 **Bias (ME and %ME) and precision (%MAE and 95CI) of pharmacogenetic and clinical algoritms by dose group in each cohort (entire, derivation and testing).** Between group comparisons calculated by paired “t” test.
(DOCX)

Table S4 **Number and percentage of correct classification (Predicted Dose** ≤**20% of Real Dose) by genetic and clinical algorithms in the derivation, validation and entire cohorts by dose group.**
(DOCX)
