# Supplementary material for: An Acenocoumarol Dosing Algorithm Using Clinical and Pharmacogenetic Data in Spanish Patients with Thromboembolic Disease
Source: PLoS One. 2012 Jul 20;7(7):e41360. doi: 10.1371/journal.pone.0041360 (PMC3401172; doi:10.1371/journal.pone.0041360)
Supplement: Table S1 — Demographic, clinical and genotype data in the model. (DOCX) [file pone.0041360.s001.docx]

**Table S1**. Demographic, clinical and genotype data in the model

| **Variable** |  |
| --- | --- |
| Gender | Male or Female |
| Age | In years |
| Body mass index (BMI) | In kg/m2 |
| Current smoker | Yes or No |
| Mini-Mental test | 0-30 |
| Patients’ education | No eduation, Primary school, Secondary school or University degree |
| Enzime inducers treatment | Yes (phenytoin, carbamazepine and rifampin) or No |
| Enzime inhibitors | Yes (azole antifungals, proton pump inhibitors and statins) or No |
| Amiodarone treatment | Yes or No |
| Non-steroidal anti-inflammatory drug | Yes or No |
| *CYP2C9* genotype |  |
| *1/*2 | Yes, No or Not Known ^(1)^ |
| *1/*3 | Yes, No or Not Known |
| *2/*2 or *2/*3 or *3/*3 | Yes, No or Not Known |
| *VKORC1* genotype |  |
| A/G | Yes or No |
| A/A | Yes or No |
| *CYP4F2* genotype |  |
| VM | Yes, No or Not Known ^(2)^ |
| MM | Yes, No or Not Known |
| *APOE* rs7412 |  |
| C/T | Yes or No |
| T/T | Yes or No |
| *APOE* rs429358 |  |
| T/C | Yes, No or Not Known ^(3)^ |
| C/C | Yes, No or Not Known |
| 1. One not known case for *CYP2C9* genotype. 2. Six not known cases for *CYP4F2* genotype 3. Three not known cases for *ApoE* rs429358 | |
