# Supplementary material for: An Acenocoumarol Dosing Algorithm Using Clinical and Pharmacogenetic Data in Spanish Patients with Thromboembolic Disease
Source: PLoS One. 2012 Jul 20;7(7):e41360. doi: 10.1371/journal.pone.0041360 (PMC3401172; doi:10.1371/journal.pone.0041360)
Supplement: Table S2 — Results of Multiple Linear Regresion (LR) in the Entire Cohort (EC) and Derivation Cohort (DC) following different methods for variable evaluation (Introduce, by forward steps and backward steps). After these analyses the variables to be included in the final model were selected as depicted in the last column. (DOCX) [file pone.0041360.s002.docx]

**Table S2.** Results of Multiple Linear Regresion (LR) in the Entire Cohort (EC) and Derivation Cohort (DC) following different methods for variable evaluation (Introduce, by forward steps and backward steps). After these analyses the variables to be included in the final model were selected as depicted in the last column.

|  | **LR EC Introduce** | **LR DC Introduce** | **LR EC Backward** | **LR DC Backward** | **LR EC Forward** | **LR DC Forward** | **Variable Selected** |
| --- | --- | --- | --- | --- | --- | --- | --- |
| **Age** | <0.001 | <0.001 | <0.001 | <0.001 | <0.001 | <0.001 | Yes |
| **Gender** | 0.111 | >0.2 | >0.2 | >0.2 | >0.2 | >0.2 | No |
| **BMI** | <0.001 | 0.001 | <0.001 | <0.001 | 0.002 | <0.001 | Yes |
| **Amiodarone** | 0.026 | 0.029 | 0.02 | .029 | 0.034 | 0.027 | Yes |
| **Statines** | >0.2 | >0.2 | >0.2 | >0.2 | >0.2 | >0.2 | No |
| **NSAIDs** | 0.097 | >0.2 | 0.085 | 0.184 | >0.2 | >0.2 | No |
| **PPI** | >0.2 | >0.2 | >0.2 | >0.2 | >0.2 | >0.2 | No |
| **Drug Inducers** | 0.042 | 0.064 | 0.053 | 0.078 | >0.2 | 0.063 | Yes |
| **Nº cigarrettes/day** | 0.11 | >0.2 | 0.048 | >0.2 | >0.2 | >0.2 | No |
| **CYP2C9 1_2** | >0.2 | >0.2 | >0.2 | >0.2 | >0.2 | >0.2 | No |
| **CYP2C9 1_3** | <0.001 | 0.001 | <0.001 | <0.001 | 0.001 | <0.001 | Yes |
| **CYP2C9 2_2** | <0.001 | <0.001 | <0.001 | <0.001 | <0.001 | <0.001 | Yes |
| **CYP2C9 Unknown** | >0.2 | >0.2 | >0.2 | >0.2 | >0.2 | >0.2 | No |
| **CYP4F2 1_2** | 0.108 | >0.2 | 0.089 | 0.158 | >0.2 | >0.2 | No |
| **CYP4F2 2_2** | <0.001 | 0.001 | <0.001 | 0.001 | 0.007 | 0.002 | Yes |
| **CYP4F2 Unknown** | 0.036 | >0.2 | 0.024 | >0.2 | >0.2 | >0.2 | No |
| **VKORC 1_2** | 0.003 | 0.036 | 0.003 | 0.053 | >0.2 | 0.037 | Yes |
| **VKORC 2_2** | <0.001 | <0.001 | <0.001 | <0.001 | >0.2 | <0.001 | Yes |
| **APOE 176 1_2** | 0.07 | >0.2 | >0.2 | >0.2 | 0.001 | >0.2 | No |
| **APOE 176 2_2** | >0.2 | 0.074 | 0.074 | 0.086 | <0.001 | 0.064 | Yes |
| **R2** | 0.618 | 0.634 | 0.607 | 0.612 | 0.531 | 0.605 |  |
