# Supplementary material for: An Acenocoumarol Dosing Algorithm Using Clinical and Pharmacogenetic Data in Spanish Patients with Thromboembolic Disease
Source: PLoS One. 2012 Jul 20;7(7):e41360. doi: 10.1371/journal.pone.0041360 (PMC3401172; doi:10.1371/journal.pone.0041360)
Supplement: Table S3 — Bias (ME and %ME) and precision (%MAE and 95CI) of pharmacogenetic and clinical algoritms by dose group in each cohort (entire, derivation and testing). Between group comparisons calculated by paired “t” test. (DOCX) [file pone.0041360.s003.docx]

**Table S3.** Bias (ME and %ME) and precision (%MAE and 95CI) of pharmacogenetic and clinical algoritms by dose group in each cohort (entire, derivation and testing). Between group comparisons calculated by paired “t” test.

|  | **Derivation Cohort** | | | | **Testing cohort** | | | | **Entire cohort** | | | |
| --- | --- | --- | --- | --- | --- | --- | --- | --- | --- | --- | --- | --- |
| **Dose Group** | **PhGx algorithm*** | **Clinical Algorithm*** | **Difference**** | **P value** | **PhGx algorithm*** | **Clinical Algorithm*** | **Difference**** | **P value** | **PhGx algorithm*** | **Clinical Algorithm*** | **Difference**** | **P value** |
| **Low** |  |  |  |  |  |  |  |  |  |  |  |  |
| ME | 2.28 (3.63) | 4.81 (3.23) | 2.53 (4.02)  1.10 to 3.95 | 0.001 | 3.61 (4.14) | 5.31 (3.64) | 1.70 (4.58)  -1.21 to 4.61 | 0.225 | 2.63 (3.77) | 4.94 (3.31) | 2.31 (4.14)  1.06 to 3.55 | 0.001 |
| MAE | 3.08 (2.95) | 4.82 (3.22) | 1.73 (3.72)  0.41 to 3.05 | 0.012 | 4.09 (3.61) | 5.31 (3.64) | 1.21 (4.30)  -1.52 to 3.95 | 0.350 | 3.36 (3.13) | 4.95 (3.30) | 1.59 (3.84)  0.44 to 2.75 | 0.008 |
| %ME | 28.53 (43.5) | 62.57 (55.24) | 34 (56.34)  14.07 to 54.03 | 0.002 | 33.69 (37.91) | 54.87 (40.69) | 21.19 (51.60)  -11,59 to 53.97 | 0.183 | 29.90 (41.75) | 60.52 (51.43) | 30.62 (54.84)  14.14 to 47.09 | 0.001 |
| %MAE | 36.96 (36.41) | 62.67 (55.13) | 25.71 (54.51)  (6.38 to 45.04) | 0.011 | 38.56 (32.45) | 54.87 (40.69) | 16.32 (49.62)  -15.21 to 47.84 | 0.279 | 37.38 (35.04) | 60.60 (51.34) | 23.20 (52.86)  7.32 to 39.08 | 0.005 |
| **Intermed.** |  |  |  |  |  |  |  |  |  |  |  |  |
| ME | -0.65 (3.86) | -0.12 (3.17) | -0.53 (2.86)  -1.33 to 0.27 | 0.187 | 1.05 (2.49) | -1.04 (2.91) | -2.10 (3.32)  -4.47 to 0.28 | 0.077 | 0.07 (3.08) | -7.72 (3.70) | -0.79 (2.97)  -1.55 to -0.29 | 0.042 |
| MAE | 2.37 (2.07) | 2.87 (2.63) | 0.50 (2.29)  -0.14 to 1.14 | 0.125 | 1.86 (1.90) | 2.49 (1.68) | 0.62 (3.18) | 0.548 | 2.28 (2.04) | 2.81 (2.49) | 0.52 (2.43)  -0.10 to 1.14 | 0.099 |
| %ME | -0.71 (20.84) | -2.25 (24.64) | -2.96 (19.80)  -8.54 to 2.61 | 0.291 | 7.87 (16.44) | -5.57 (17.64) | -13.45 (20.71)  -28.26 to 1.37 | 0.07 | 1.88 (20.24) | -2.79 (23.54) | -4.68 (20.18)  -9.85 to 0.49 | 0.075 |
| %MAE | 15.36 (13.94) | 17.86 (16.93) | 2.50 (15.50)  -1.87 to 6.87 | 0.256 | 12.59 (12.77) | 15.26 (9.33) | 2.66 (19.57)  -11.33 to 16.66 | 0.677 | 14.91 (13.69) | 17.43 (15.91) | 2.53 (16.09)  -1.59 to 6.65 | 0.225 |
| **High** |  |  |  |  |  |  |  |  |  |  |  |  |
| ME | -4.69 (6.15) | -8.55 (6.13) | -3.86 (2.87  -4.92 to -2.82 | <0.001 | -5.58 (3.05) | -7.14 (1.75) | -1.56 (0.78)  -3.4 to 0.29 | 0.086 | -4.87 (5.63) | -8.26 (5.53) | -3.38 (2.88)  -4.32 to -2.45 | <0,001 |
| MAE | 6.27 (4.46) | 9.00 (5.41) | 2.70 (3.26)  1.54 to 3.93 | <0.001 | -5.58 (3.05) | -7.14 (1.75) | -1.56 (2.21)  -0.29 to 3.40 | 0.086 | 6.13 (4.18) | 8.62 (4.93) | 2.49 (3.08)  1.50 to 3.49 | <0,001 |
| %ME | -15.12 (22.03) | -29.83 (19.48) | -14.71 (10.85)  -18.69 to -10.73 | <0.001 | -23.02 (12.18) | -30.05 (7.76) | -7.03 (9.67)  -15.12 to 1.05 | 0.079 | -16.74 (20.51) | -29.87 (17.63) | -13.13 (10.96)  -16.68 to -9.58 | <0,001 |
| %MAE | 22.30 (14,40) | 32.00 (15.53) | 9.69 (12.45)  5.13 to 14.26 | <0.001 | 23.02 (12.18) | 30.05 (7.76) | 7.03 (9.67)  -1.05 to 15.12 | 0.079 | 22.45 (13.83) | 31.60 (14.21) | 9.14 (11.87)  5.30 to 13.00 | <0,001 |

***** Mean (standard deviation); ** Mean (standard deviation) and 95% Confidence Interval
