# Supplementary material for: An Acenocoumarol Dosing Algorithm Using Clinical and Pharmacogenetic Data in Spanish Patients with Thromboembolic Disease
Source: PLoS One. 2012 Jul 20;7(7):e41360. doi: 10.1371/journal.pone.0041360 (PMC3401172; doi:10.1371/journal.pone.0041360)
Supplement: Table S4 — Number and percentage of correct classification (Predicted Dose ≤20% of Real Dose) by genetic and clinical algorithms in the derivation, validation and entire cohorts by dose group. (DOCX) [file pone.0041360.s004.docx]

**Table S4.** Number and percentage of correct classification (Predicted Dose ≤ 20% of Real Dose) by genetic and clinical algorithms in the derivation, validation and entire cohorts by dose group

|  | **Low Doses** | | | **Median Doses** | | | **High Doses** | | |
| --- | --- | --- | --- | --- | --- | --- | --- | --- | --- |
|  | **PhGx** | **Clinical** | **ARR** | **PhGx** | **Clinical** | **ARR** | **PhGx** | **Clinical** | **ARR** |
| **Derivation Cohort (n=117)** | 15/34  44.1% | 5/34  14.7% | 29*  (9-50) | 41/52  78.9% | 32/52  61.5% | 17*  (0.0-35) | 14/31  45.2% | 7/31  22.6% | 23*  (0.0-45) |
| **Testing cohort (n=30)** | 4/12  33.3% | 1/12  8.3% | 25  (-6 – 56) | 7/10  70.0% | 6/10  60.0% | 10  (-31 –52) | 3/8  37.5% | 0/8  0.0% | 38*  (4-71) |
| **Entire Cohort (n=147)** | 19/46  41.0% | 6/46  13.0% | 29*  (11-46) | 48/62  77.0% | 38/62  61.0% | 16*  (0-32) | 17/39  44.0% | 7/39  18.0% | 26*  (6-45) |

* p< 0.05 for the comparison of the Pharmacogenetic vs Clinical algoritm.
